# Legionella associated rhabdomyolysis: a case report

**DOI:** 10.1186/s13256-023-04000-1

**Published:** 2023-06-22

**Authors:** Helena Ma, Avni Bavishi, Bijal Jain

**Affiliations:** 1 Northwestern University Feinberg School of Medicine, 420 E. Superior St, Chicago, IL 60611 USA; 2 Department of Medicine, Northwestern Memorial Hospital, 251 E Huron St, Chicago, IL 60611 USA; 3 Department of Medicine, Jesse Brown VA Medical Center, 820 S Damen Ave, Chicago, IL 60612 USA

**Keywords:** Legionnaire’s disease, Rhabdomyolysis, Infectious causes, Renal impairment, Case report

## Abstract

**Background:**

Infections have been recognized as an uncommon cause of rhabdomyolysis, with evidence indicating a worse prognosis when compared to rhabdomyolysis caused by other etiologies. Diseases caused by *Legionella pneumophila* can present variably, ranging from mild to severe illness, as is sometimes the case with pneumonia. In particular, the triad of Legionnaire’s disease, rhabdomyolysis, and acute kidney injury is associated with a significant increase in the morbidity and mortality, with most patients requiring initiation of renal replacement therapy such as hemodialysis. While the exact mechanism of both the muscle and kidney injury in this setting remains unknown, several hypotheses exist, with some research suggesting multiple yet distinct processes occurring in both target organs.

**Case presentation:**

In this case report, we describe a 53-year-old African American man who presented with *Legionella pneumophila* pneumonia complicated by rhabdomyolysis and acute kidney injury. He was treated with aggressive fluid resuscitation and a 2-week course of azithromycin. His clinical status improved without necessitating renal replacement therapy or mechanical ventilation. We postulate that early recognition and treatment were key to his recovery. He was discharged 10 days later without recurrence of rhabdomyolysis at the time of this report.

**Conclusion:**

While there are several well-established and more common causes of rhabdomyolysis, clinicians should recognize *Legionella sp.* as an etiology, given its association with significant morbidity and mortality.

## Background

*Legionella pneumophila* is a gram-negative bacteria, often found in freshwater such as lakes and rivers with the ability to proliferate in human made water systems [1]. Legionella infection typically causes a self-limiting acute febrile illness known as Pontiac fever. A more severe form of the infection causes pneumonia, called Legionnaire’s disease, which has a case fatality rate of 10%. The variable presentations of Legionnaire’s disease can make diagnosis a challenge, prompting the development of a clinical prediction tool (CPT) to aid in diagnosis. The tool utilizes clinical features such as elevated body temperature, absence of sputum, low serum sodium, elevated LDH and CRP, and low platelet counts. According to the CPT, fewer than two features reliably rules out Legionella infection with a negative predictive value of 99.4% [2].

Rhabdomyolysis is the clinical syndrome of muscle breakdown resulting in the release of muscle proteins and enzymes into the bloodstream. Myoglobin, a heme-containing protein, is nephrotoxic and responsible for the acute kidney injury often seen with rhabdomyolysis. It is most commonly associated with trauma, crush injuries, medications, toxins, and electrolyte disturbances [3]. However, approximately 5% of adult rhabdomyolysis cases have infectious causes, including *Legionella pneumophila,* Streptococcus spp*.,* HIV, and Influenza [4].

In this case report, we discuss a patient who presented with Legionnaire’s disease, rhabdomyolysis, and acute kidney injury. This triad, while uncommon, is associated with up to a 40% increased mortality of Legionnaire’s disease [5] and the need for renal replacement therapy in a majority of patients [6]. Accordingly, we advise clinicians to consider infectious causes in their differential diagnosis for rhabdomyolysis, as early diagnosis and appropriate treatment can potentially improve outcomes. We will also review proposed mechanisms for infectious causes of rhabdomyolysis and acute kidney injury, along with key management strategies.

## Case presentation

A 53-year old African American man with a past medical history of untreated asthma and polysubstance use presented to our hospital with a 3-day history of shortness of breath, subjective fevers, and chills. He also reported generalized lethargy and diffuse muscle fatigue without muscle pain. The patient denied any sick contacts or recent travel. He endorsed regular use of tobacco, cocaine, alcohol, and cannabis up until hospital admission, with no increase in quantity in the preceding days. On further history, he also noted that he recently moved into a new home that required him to clean a basement bathtub filled with dirty standing water. It was shortly after this activity that the patient recalled developing fatigue, which then progressed to his presenting complaints.

On admission, the patient was afebrile (97.2 ℉), tachycardiac (heart rate 130) and tachypneic (respiratory rate of 22). Shortly after admission, he developed a fever to 102 ℉ (38.9 ℃). His oxygen saturations ranged from 86 to 88% on room air, improving to 94–96% with 2L of supplemental oxygen by nasal cannula. He was lethargic but arousable and appeared disheveled and dehydrated. Cardiac auscultation was unremarkable without any pathological murmurs. Auscultation of the lungs revealed bilateral wheezes with decreased breath sounds and crackles throughout the left posterior lung zone. No jugular venous distention or lower limb swelling were appreciated. Abdominal exam was unremarkable with no distension, guarding, rebound, rigidity or tenderness. Neurological exam showed 4/5 strength in his upper and lower extremities with intact movement, reflexes, and sensation.

**Fig. 1:**
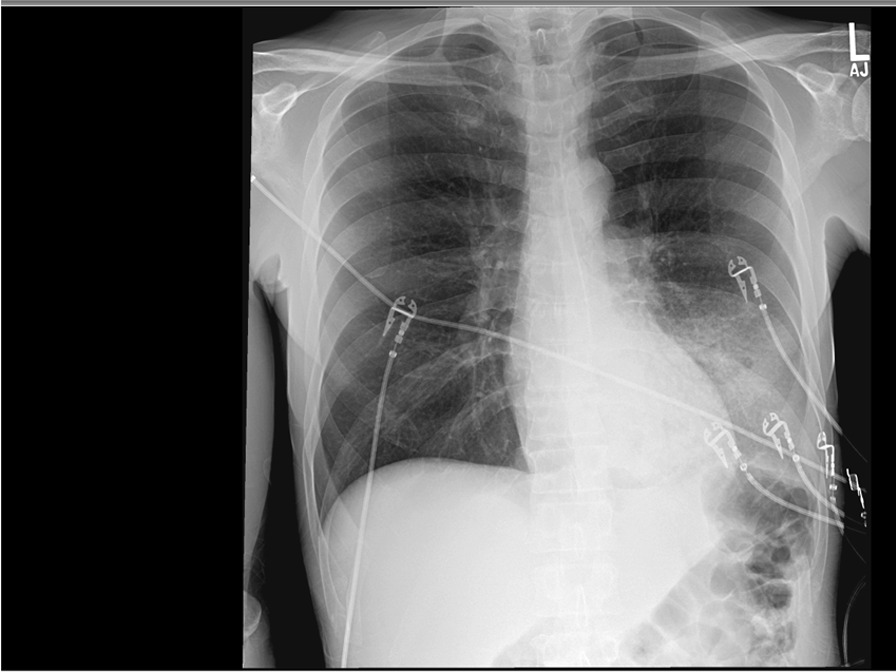
Chest X-ray on admission showing dense left lower lobe consolidation

Laboratory tests on admission revealed a white cell count of 12.1 × 10^9^/L with 80% PMNs and 17% bands, hemoglobin concentration of 15.5 g/dL, procalcitonin of 34.3 ng/mL, sodium of 128 mmol/L, potassium of 3.9 mmol/L, calcium of 8.1 mmol/L, glucose of 246 mg/dL, blood urea nitrogen (BUN) of 75 mg/dL, creatinine of 3.32 mg/dL (baseline 0.7 mg/dL), alanine aminotransferase (ALT) of 496 U/L, aspartate aminotransferase (AST) of 1393 U/L, and lactate dehydrogenase (LDH) of 412 U/L. Urinalysis showed large blood with 2–3 red blood cells on reflex microscopy. Urine toxicology was positive for cannabinoids and cocaine. Serum creatine kinase level resulted as > 25, 000 U/L. Chest X-ray demonstrated left lower lobe consolidation (Fig. 1). Electrocardiogram showed sinus tachycardia.

The patient was diagnosed with sepsis due to community acquired pneumonia, complicated by hypoxemic respiratory failure, and acute kidney injury likely due to rhabdomyolysis. Legionella infection was considered high on the differential given his hyponatremia, high LDH, elevated hepatic transaminases, confusion, and potential exposure to contaminated water source. He was promptly started on intravenous fluids at 125 cc/hour as well as antibiotic therapy consisting of IV azithromycin (500 mg daily) and IV ceftriaxone (1 g daily).

On hospital days 2–3, infectious studies resulted and demonstrated no growth on sputum culture and a negative *Streptococcus pneumoniae* urinary antigen test. The *Legionella pneumophila* urinary antigen test was positive. Respiratory viral panel was negative for all pathogens, including SARS-COV-2. Blood culture showed no growth throughout the hospitalization. Ceftriaxone was discontinued and the azithromycin course was extended for a total of 14 days (PO azithromycin 500 mg daily).

**Fig. 2:**
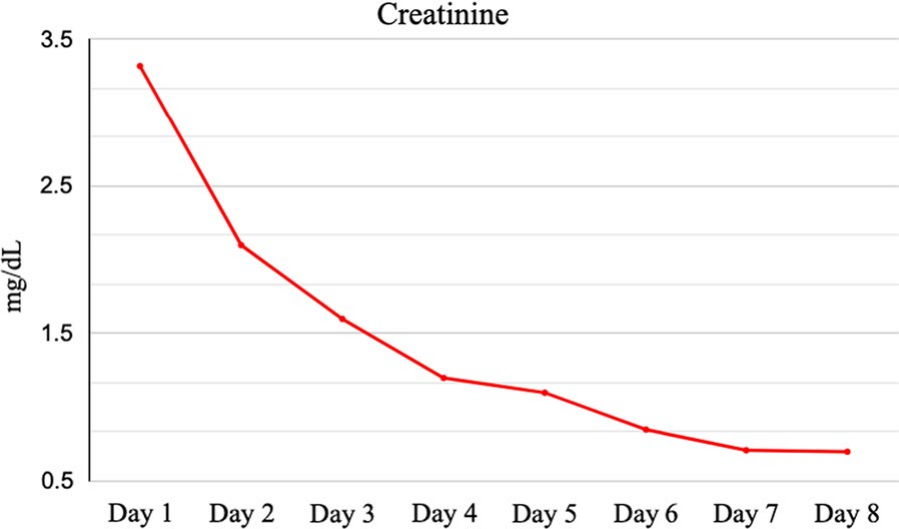
Serum creatinine trend during hospital stay. Creatinine level was elevated at 3.32 mg/dL on admission and gradually stabilized to a baseline of 0.7 mg/dL by discharge

**Fig. 3:**
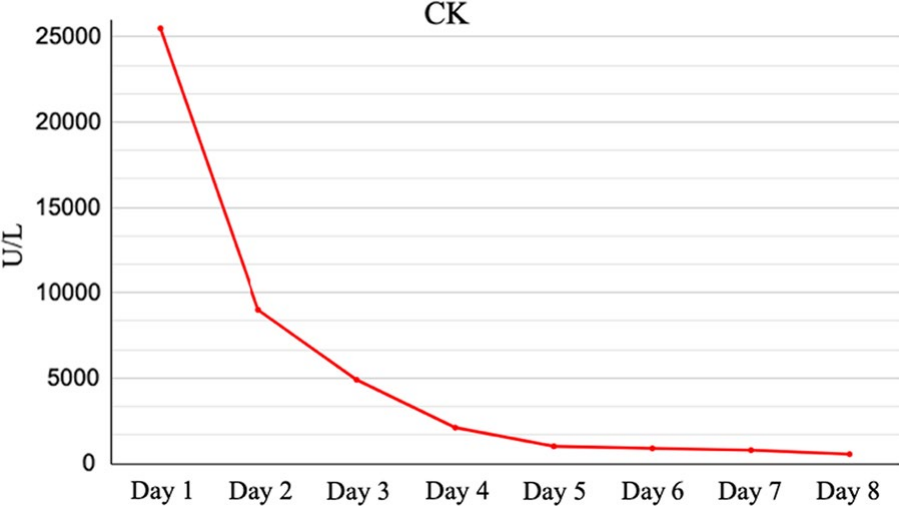
Serum creatinine kinase trends during hospital stay. CK level was elevated at 25,493 U/L on admission and improved to 542 U/L by discharge

While the patient’s white blood cell count, serum creatinine, creatine kinase, AST and ALTs steadily downtrended from the time of admission (Figs. 2, 3), he continued to be febrile, tachycardic, tachypneic, and hypoxic through day 4 of hospitalization. A thorough evaluation for secondary infections or complications related to Legionella pneumonia was unrevealing. The patient finally started clinically improving day 5 and onwards. He did not require invasive ventilation or hemodialysis. On hospital day 10, renal function returned to baseline and the patient was no longer requiring oxygen. He was discharged home with instructions to complete the course of azithromycin. As of one year post discharge, the patient has not had recurrence of rhabdomyolysis.

## Discussion

This case highlights the deadly triad of Legionella, rhabdomyolysis, and acute kidney injury, and how mortality and morbidity can likely be decreased with early detection and aggressive management. While many patients with this deadly triad have an extended hospital stay and often need dialysis, our patient never required dialysis, invasive ventilation, or ICU management. Furthermore, he was able to be discharged from the hospital after only 10 days of inpatient management with no recurrence of rhabdomyolysis in the year following discharge. This positive outcome emphasizes the importance of early detection, atypical antibiotic coverage, and aggressive fluid management for treating cases like this.

Rhabdomyolysis is characterized by muscle breakdown and release of myoglobin and creatine kinase into the bloodstream. The classic symptoms associated with rhabdomyolysis are muscle pain, weakness, and dark urine [7]. However, less than 10% of patients develop this triad [3] and more than half of patients may not report any muscular symptoms [8]. Laboratory evaluation classically demonstrates significantly elevated serum CK levels and large blood on urine dipstick (due to myoglobin) with few to no red blood cells on microscopic evaluation. Electrolyte derangements are common as are elevated hepatic transaminases, with AST predominance due to the breakdown of skeletal muscle. Acute kidney injury can develop in up to 50% of patients [9].

Common causes of rhabdomyolysis include trauma, crush injuries, metabolic myopathies, electrolyte imbalances, toxins and medications. Infectious causes of rhabdomyolysis are less common, but are still important causes to consider in the differential for rhabdomyolysis. Influenza is the most common viral etiology followed by HIV and enterovirus [4]. Legionella is the most common bacterial etiology followed by Streptococcus, Francisella, and Salmonella [4]. Other infectious causes are listed in Table 1.

**Table 1:** Infectious causes of rhabdomyolysis

Viral causes	● Influenza ● HIV ● Enterovirus ● Epstein-Barr virus ● Herpes simplex virus ● Coxsackievirus ● Adenovirus ● Parainfluenza ● Echovirus ● Varicella-zoster virus ● Cytomegalovirus ● SARS-CoV-2
Bacterial causes	● *Legionella* species ● *Streptococcus pneumoniae* ● *Streptococcus agalactiae* ● *Streptococcus pyogenes* ● *Viridans streptococci* ● *Francisella tularensis* ● *Salmonella* species ● *Staphylococcus epidermidis* ● *E. coli* ● *Leptospira* species ● *Coxiella* ● *Listeria* species ● *Vibrio* species ● *Brucella* species ● *Bacillus* species ● *Borrelia burgdorferi* ● *Clostridium perfringens* ● *Klebsiella species*
Other infectious causes	● *Candida* species ● *Aspergillus* species ● *Mycoplasma pneumoniae* ● *Plasmodium* species

Reference: [6, 10, 11]; [3, 12]

*E. coli* Escherichia coli, *SARS-CoV-2* Severe acute respiratory syndrome coronavirus 2, *HIV* Human immunodeficiency virus

Bacterial causes are associated with significant morbidity and mortality, with 57% of the cases reporting acute renal failure and 38% resulting in death [4]. Despite being the most common bacterial etiology, our understanding of Legionella-associated rhabdomyolysis complicated by acute kidney injury is very limited due to the low numbers of published case reports since 1980 [6]. Several mechanisms have been proposed to explain how different infections cause rhabdomyolysis, though in many cases the exact process remains unknown.

In regards to viral infections, hypotheses include direct invasion of muscle tissue, myotoxic cytokines, viral toxins, and immune-mediated processes [13, 14]. For example, direct invasion of muscle tissue by the influenza virus has been shown on pathologic examination in some case reports [15], but this has not been reported consistently. Another possibility includes the release of myotoxic cytokines. In another case report, a patient who developed coxsackie associated rhabdomyolysis was found to have an elevated level of serum tumor necrosis factor (TNF), which has been shown to induce skeletal muscle breakdown in an animal model [16]. Finally, because of the inability to isolate the virus in some settings, other researchers have suggested that immune-mediated processes might be responsible for the muscle injury, though the specifics remain unknown [14, 15].

Similarly, the pathogenesis for bacteria-induced rhabdomyolysis is poorly understood. There is debate whether the rhabdomyolysis is primarily due to the bacterial organism versus resulting from secondary effects of infection (rigors, tissue hypoxia due to sepsis and dehydration). For Legionella specifically, two possible mechanisms have been proposed: direct invasion of Legionella into the muscle itself or the release of endotoxin into the circulation with subsequent muscle injury. Legionella has been identified occasionally in organs other than the lung, a finding that suggests that direct bacterial involvement might be causing the systemic abnormality associated with Legionnaire’s disease [17]. In fact, there have been a small number of cases where the *Legionella pneumophila* was isolated from peripheral blood samples, demonstrating that the bacteria may be disseminated hematologically [18–20]. In contrast, there have also been cases reporting on the absence of Legionella immunofluorescence on muscle biopsy [22, 23], with one study also noting an absence of inflammatory infiltrates in the muscle, arguing against direct invasion by the organism [21]. Finally, there is a hypothesis suggesting circulating endotoxins as the cause for Legionella-induced rhabdomyolysis and other systemic manifestations of this disease [5]. The proposed mechanism is that the endotoxin may have a vasoconstrictive effect on small blood vessels leading to local ischemia-induced changes [24].

As for the mechanism of acute kidney injury, both direct and indirect causes have been implicated. Due to the nephrotoxic properties of myoglobin compounded by the frequent occurrence of rhabdomyolysis in clinical settings associated with renal hypoperfusion, one plausible explanation is the combined effect of hemodynamic and toxic insults to the kidney [6]. There are also reports suggesting direct invasion by Legionella as the mechanism of kidney injury [5]. Finally, in our patient’s case, cocaine use was likely a contributing factor as cocaine has been found to be associated with rhabdomyolysis due to increased sympathomimetic activity and arterial vasoconstriction that can cause skeletal muscle ischemia and infarction. Additionally, cannabis has been rarely associated with rhabdomyolysis. However, we do not believe cocaine or cannabis was the main cause of his rhabdomyolysis given the fact he was a regular user with no recent increase in consumption.

The key management strategy for acute kidney injury resulting from rhabdomyolysis is aggressive fluid resuscitation in order to compensate for fluid sequestration in the muscles in addition to promoting renal filtration [3]. The treatment for community acquired pneumonia is typically a 3 to 5-day course of quinolones (as monotherapy) or a combination of macrolide and a beta-lactam. Certain infections, such as Legionella, can require a longer course, up to 2 weeks according to Infectious Diseases Society of America guidelines [25]. Additionally, the preferred antibiotics for Legionnaire’s disease are azithromycin or a fluoroquinolone, highlighting the importance of empiric coverage for atypical pathogens while cultures or Legionella-specific tests are pending. In our patient’s case, we initially suspected that the rhabdomyolysis was due to fevers/sepsis, immobility, and cocaine use. However, literature review revealed an association between Legionella and rhabdomyolysis and that the triad of Legionnaire’s disease, rhabdomyolysis, and acute kidney injury is associated with a significant increase in morbidity and mortality. We believe that early diagnosis and appreciation of the associated increased risk influenced our management strategy and likely contributed to the positive outcome.

## Conclusion

Clinicians should consider infections, such as Legionella, as an etiology in the work up of rhabdomyolysis, given the documented association with significant morbidity and mortality, and potential benefit of early detection and management. The triad of Legionnaire’s disease, rhabdomyolysis, and renal failure is of particular concern since it has been associated with a 40% increase in mortality. Key management involves early detection of Legionella with a urinary test and early initiation of appropriate antibiotics such as azithromycin or quinolones and aggressive intravenous fluid resuscitation. This case also highlights the importance of empiric antimicrobial coverage for atypical organisms, such as *Legionella pneumophilia*, in patients presenting with pneumonia and sepsis.

## Data Availability

Not applicable.
